# Frequency distribution

**DOI:** 10.4103/0976-500X.77120

**Published:** 2011

**Authors:** S Manikandan

**Affiliations:** *Assistant Editor, JPP*

## INTRODUCTION

The next step after the completion of data collection is to organize the data into a meaningful form so that a trend, if any, emerging out of the data can be seen easily. One of the common methods for organizing data is to construct frequency distribution. Frequency distribution is an organized tabulation/graphical representation of the number of individuals in each category on the scale of measurement.[1] It allows the researcher to have a glance at the entire data conveniently. It shows whether the observations are high or low and also whether they are concentrated in one area or spread out across the entire scale. Thus, frequency distribution presents a picture of how the individual observations are distributed in the measurement scale.

## DISPLAYING FREQUENCY DISTRIBUTIONS

### Frequency tables

A frequency (distribution) table shows the different measurement categories and the number of observations in each category. Before constructing a frequency table, one should have an idea about the range (minimum and maximum values). The range is divided into arbitrary intervals called “class interval.” If the class intervals are too many, then there will be no reduction in the bulkiness of data and minor deviations also become noticeable. On the other hand, if they are very few, then the shape of the distribution itself cannot be determined. Generally, 6–14 intervals are adequate.[2]

The width of the class can be determined by dividing the range of observations by the number of classes. The following are some guidelines regarding class widths:[1]

- It is advisable to have equal class widths. Unequal class widths should be used only when large gaps exist in data.
- The class intervals should be mutually exclusive and nonoverlapping.
- Open-ended classes at the lower and upper side (e.g., <10, >100) should be avoided.

The frequency distribution table of the resting pulse rate in healthy individuals is given in Table 1. It also gives the cumulative and relative frequency that helps to interpret the data more easily.

**Table 1:** Frequency distribution of the resting pulse rate in healthy volunteers (N = 63)

Pulse/min	Frequency	Cumulative frequency	Relative cumulative frequency (%)
60–64	2	2	3.17
65–69	7	9	14.29
70–74	11	20	31.75
75–79	15	35	55.56
80–84	10	45	71.43
85–89	9	54	85.71
90–94	6	60	95.24
95–99	3	63	100

### Frequency distribution graphs

A frequency distribution graph is a diagrammatic illustration of the information in the frequency table.

#### Histogram

**Figure 1:**
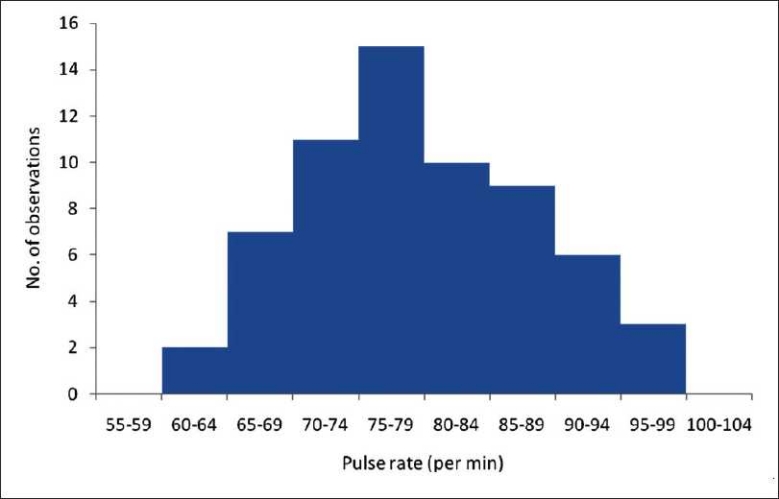
Histogram of the resting pulse rate in healthy volunteers (N = 63)

A histogram is a graphical representation of the variable of interest in the *X* axis and the number of observations (frequency) in the *Y* axis. Percentages can be used if the objective is to compare two histograms having different number of subjects. A histogram is used to depict the frequency when data are measured on an interval or a ratio scale. Figure 1 depicts a histogram constructed for the data given in Table 1.

A bar diagram and a histogram may look the same but there are three important differences between them:[3,4]

In a histogram, there is no gap between the bars as the variable is continuous. A bar diagram will have space between the bars.

All the bars need not be of equal width in a histogram (depends on the class interval), whereas they are equal in a bar diagram.

The area of each bar corresponds to the frequency in a histogram whereas in a bar diagram, it is the height [Figure 1].

#### Frequency polygon

**Figure 2:**
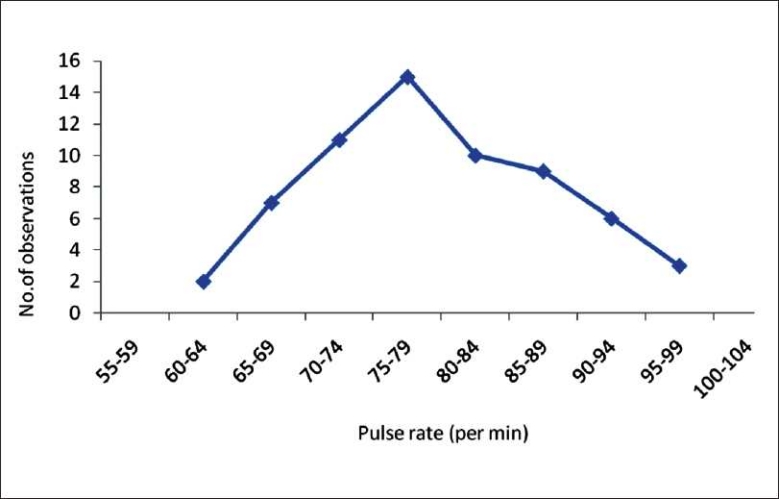
Frequency polygon of the resting pulse rate in healthy volunteers (N = 63)

A frequency polygon is constructed by connecting all midpoints of the top of the bars in a histogram by a straight line without displaying the bars. A frequency polygon aids in the easy comparison of two frequency distributions. When the total frequency is large and the class intervals are narrow, the frequency polygon becomes a smooth curve known as the frequency curve. A frequency polygon illustrating the data in Table 1 is shown in Figure 2.

#### Box and whisker plot

This graph, first described by Tukey in 1977, can also be used to illustrate the distribution of data. There is a vertical or horizontal rectangle (box), the ends of which correspond to the upper and lower quartiles (75^th^ and 25^th^ percentile, respectively). Hence the middle 50% of observations are represented by the box. The length of the box indicates the variability of the data. The line inside the box denotes the median (sometimes marked as a plus sign). The position of the median indicates whether the data are skewed or not. If the median is closer to the upper quartile, then they are negatively skewed and if it is near the lower quartile, then positively skewed.

**Figure 3:**
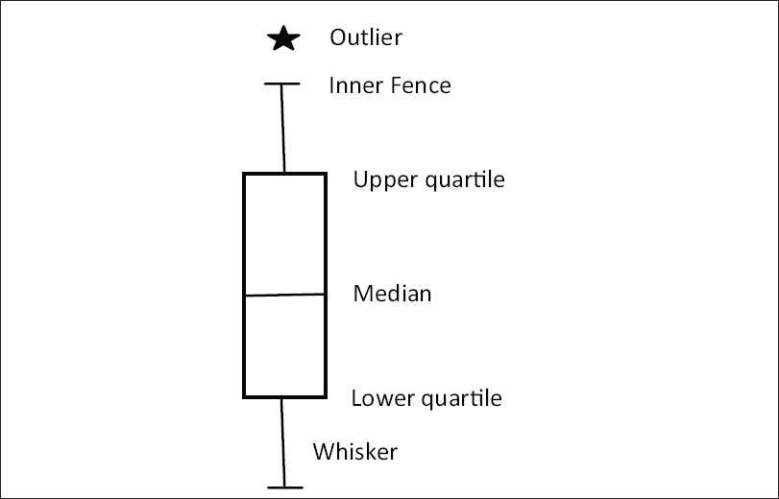
Schematic diagram of a “box and whisker plot”

The lines outside the box on either side are known as whiskers [Figure 3]. These whiskers are 1.5 times the length of the box, i.e., the interquartile range (IQR). The end of whiskers is called the inner fence and any value outside it is an outlier. If the distribution is symmetrical, then the whiskers are of equal length. If the data are sparse on one side, the corresponding side whisker will be short. The outer fence (usually not marked) is at a distance of three times the IQR on either side of the box. The reason behind having the inner and outer fence at 1.5 and 3 times the IQR, respectively, is the fact that 95% of observations fall within 1.5 times the IQR, and it is 99% for 3 times the IQR.[5]

## CHARACTERISTICS OF FREQUENCY DISTRIBUTION

There are four important characteristics of frequency distribution.[6] They are as follows:

- Measures of central tendency and location (mean, median, mode)
- Measures of dispersion (range, variance, standard deviation)
- The extent of symmetry/asymmetry (skewness)
- The flatness or peakedness (kurtosis).

These will be dealt with in detail in the next issue.
